# Nobel Prizes: Contributions to Cardiology

**DOI:** 10.5935/abc.20150041

**Published:** 2015-08

**Authors:** Evandro Tinoco Mesquita, Luana de Decco Marchese, Danielle Warol Dias, Andressa Brasil Barbeito, Jonathan Costa Gomes, Maria Clara Soares Muradas, Pedro Gemal Lanzieri, Ronaldo Altenburg Gismondi

**Affiliations:** Universidade Federal Fluminense, Niterói, RJ – Brazil

**Keywords:** Cardiology, Nobel Prize, History, Cardiovascular Diseases / trends

## Abstract

The Nobel Prize was created by Alfred Nobel. The first prize was awarded in 1901 and Emil Adolf von Behring was the first laureate in medicine due to his research in diphtheria serum. Regarding cardiology, Nobel Prize’s history permits a global comprehension of progress in pathophysiology, diagnosis and therapeutics of various cardiac diseases in last 120 years. The objective of this study was to review the major scientific discoveries contemplated by Nobel Prizes that contributed to cardiology. In addition, we also hypothesized why Carlos Chagas, one of our most important scientists, did not win the prize in two occasions. We carried out a non-systematic review of Nobel Prize winners, selecting the main studies relevant to heart diseaseamong the laureates. In the period between 1901 and 2013, 204 researches and 104 prizes were awarded in Nobel Prize, of which 16 (15%) studies were important for cardiovascular area. There were 33 (16%) laureates, and two (6%) were women. Fourteen (42%) were American, 15 (45%) Europeans and four (13%) were from other countries. There was only one winner born in Brazil, Peter Medawar, whose career was all in England. Reviewing the history of the Nobel Prize in physiology or medicine area made possible to identify which researchers and studies had contributed to advances in the diagnosis, prevention and treatment of cardiovascular diseases. Most winners were North Americans and Europeans, and male.

## Introduction

**Figure 1:**
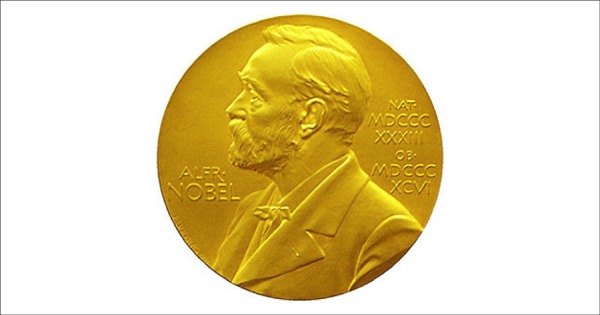
Nobel Prize Logo.

Source: http://en.wikipedia.org/wiki/Nobel_Prize

The Nobel Prize was created by Alfred Nobel (1833-1896) (Figure 1)^1,2^. In his will, the Swedish researcher determined the creation of a foundation that would carry his name, of which main objective would be to reward every year, individuals that provided outstanding contributions to mankind in the areas of Peace/Diplomacy, Literature, Chemistry, Physics and Physiology/Medicine. The first prizes were awarded in 1901 and the first winner in Medicine was Emil Adolf von Behring, for his work with diphtheria serum^1^. Since that date, there have been 876 Nobel Prize winners, of which 104 awards and 204 researchers in the area of Medicine or Physiology^1^.

The objective of the present study was to review the major scientific discoveries that received the Nobel Prize and directly or indirectly contributed to advances in physiopathology, diagnosis and treatment of cardiovascular diseases.

## Methods

We performed a systematic search of the main non Nobel Prize winners from 1901 to 2013. The winners list was obtained from the Nobel Prize site^3^ and, subsequently, information about the authors and their research were obtained from the Medline/PubMed database. Moreover, due to the specific nature of the research involving historical and bibliographical data, the Google search engine was utilized, using as descriptors the names of the researchers awarded the Nobel Prize. The results are displayed in ascending chronological order.

## Results

### A brief history of the Nobel Prize

For many historians, the interest of Alfred Nobel in medicine derived from a poor health^2^. There are reports of dyspepsia, headaches and bouts of depression. In adulthood, he would have suffered from coronary artery disease, with frequent episodes of angina. His doctors recommended the use of nitroglycerin, a substance Nobel manipulated in the explosives industry, but he would have refused. In later life he had a stroke and had to live with motor sequelae. Skeptical and suspicious, Nobel expressed in his will the wish that after his death, his veins were "open" and the signs of death confirmed by "competent doctors," before the body was sent to be cremated^1^.

Nobel’s choice of the Karolinska Institute in his will surprised many scientists. This institute was created in 1810 from the merging of a Medical School and a small surgical center where barbers were trained to perform amputations. For years it did not have the status of School of Medicine and depended on a contract with the University of Uppsala, in Sweden, for the training of its professionals^1^. Currently, the institute is one of the leading Assistance Medicine and Research centers in Europe. Five Nobel Prize winners over the last 120 years, came from this institute^1^.

### The Nobel Prize and Cardiology

In the area of cardiology, specifically regarding heart failure (HF), knowledge of the history of the Nobel Prize helps to understand important diagnostic and therapeutic advances made in the last 120 years (Chart 1). The first laureate in this area was Alexis Carrel, for his discoveries related to blood vessel suture, an important step in the further development of cardiac surgery^1,3^. Only one Brazilian, who lived in England, was awarded. His name is Peter Medawar and he has carried out researches in the immunosuppression area, with future applicability in renal and cardiac transplant^4,5^.

**Chart 1:** Main scientific discoveries that received the Nobel Prize in Physiology or Medicine related to the Cardiology area

**Year**	**Author**	**Prize**
1912	Alexis Carrel	Work on vascular suture and transplant of blood vessels and organs
1924	Willem Einthoven	Electrocardiogram
1953	Hans Adolf Krebs	Citric acid cycle (Krebs cycle)
1956	Werner Forssmann, Andre Cournard and Dickinson W. Richards	Cardiac catheterization
1960	Frank Burnet and Peter Medawar	Discovery of the immunological tolerance mechanism
1964	Konrad Bloch and Feodor Lynen	Understanding of cholesterol metabolism
1979	Allan Cormack and Godfrey Hounsfield	Computed tomography techniques
1982	Bengt Samuelsson, Sune Bergström and John Vane	"Discovery" of the angiotensin-converting enzyme inhibitors
1985	Michael Brown and Joseph Goldstein	Discovery of LDL-cholesterol receptors
1988	James Black, Gertrude Elion and George Hitchings	Development of beta-blockers
1990	Joseph Edward Murray and Edward Thomas	Development of organ and tissue transplant
1998	Robert Furchgott, Ferid Murad and Louis Ignarro	Discoveries about nitric oxide
2003	Paul Lauterbur and Peter Mansfield	Magnetic Resonance

LDL: low-density lipoprotein.

Although Carlos Chagas researched and discovered the etiological agent, the vector, the mode of transmission and clinical forms of trypanosomiasis, a unique feat in the history of world’s science, he was a candidate for the Nobel a few times, but was unsuccessful. There are hypotheses that attempt to explain why a genuinely Brazilian contribution in the field of cardiology has not been contemplated with the Nobel prize^6,7^.

### Major studies important for Cardiology that were awarded the Nobel Prize

#### Alexis Carrel – 1912: blood vessel suture

Alexis Carrel, was born in Sainte-Foy-lès-Lyon, France, graduated in Medicine from the School of Medicine of Lyon in 1893 and completed his doctorate in 1900, with a research on blood vessel sutures. He taught Anatomy and Surgery at the University of Lyons and moved to the United States in 1904, where he worked at the University of Chicago. Later, he joined the Rockefeller Institute for Medical Research in New York, where he performed most of the experiments that led to the Nobel Prize in Physiology or Medicine in 1912. He served the medical corps of the French Army during World War I (1914 -1918), where he successfully used the Carrel-Dakin method of constant irrigation of wounds with antiseptic solution, which decreased cases of death and amputations^1-3,8^.

His early work was on surgical techniques in blood vessels and arteriovenous anastomoses. After 1908, he developed methods for organ cryopreservation and transplantation^1-3,8^. In 1935, he created a system for sterile oxygen supply and preservation of organs removed from the body. He also cooperated with other researchers for the development of cardiac valvotomy surgery and sarcoma cell culture. He published the books “The culture of organs” and “Treatment of infected wounds”^1-3,8^. The suture of blood vessels was essential for the development of vascular surgery in later years.

#### Willem Einthoven – 1924: The Electrocardiogram

Willem Einthoven was born on May 21, 1860 on the island of Java, now Indonesia, and moved to the Netherlands in 1870, where he graduated from Medical School at the University of Utrecht, one of the oldest and most traditional medical schools in Europe. He was a physiologist and in the early 20^th^ century, he published several papers on the use of the galvanometer in the recording of the human electrocardiogram (ECG), which served as the basis for the current devices. The original equipment weighed more than 270 kg and was operated by several people in his laboratory. He described the P, QRS and T waves, as well as and their alterations in several diseases, especially in valvular heart disease. Showing great wisdom, he wrote after his initial research, “the electrocardiogram should not be used, at least exclusively, to diagnose valvular alterations, because the ECG is the expression of the heart muscle contraction and changes only to the extent that a valvular function failure has influence on such contraction”^1,2,9^.

In addition to the ECG, Einthoven played an important role in the development of the phonocardiogram^1,2,9^. This device, now a museum piece in many medical schools, was crucial in the supplementary examination to medical workup. Before the invention of echocardiography, many valvular heart disease diagnoses were attained by auscultation and the phonocardiogram. The valvular diseases were, until the early 20^th^ century, one of the most important causes of heart failure, particularly those of rheumatic origin.

#### Hans Adolf Krebs – 1953: Krebs cycle

Hans Adolf Krebs was born on August 25, 1900, in Hildeshiem, northern Germany^10^. He served as a military in the German army in World War I. Although he graduated in Medicine, he had to go into exile and left Germany in the 1930s, because of his Jewish descent. He chose to live in England, where he developed his research related to cell physiology. He described, together with other researchers, the urea cycle, the citric acid cycle, and especially, cellular respiration, which produces adenosine triphosphate (ATP) from glucose and oxygen^10,11^. In his honor, this sequence of biochemical reactions is known as "Krebs cycle".

It has long been known that mitochondrial diseases are a group of diseases related to neuromuscular dysfunction and cardiomyopathy^12,13^. Moreover, in cardiology, the study of the mitochondrial function in the myocyte is a new research frontier, which some have called "mitochondrial bioenergetics"^12,13^. One of the key areas is HF, due to the role of aerobic metabolism in myocardial performance. Drugs are being developed that act on mitochondrial pathways, correcting occasional dysfunctions, and it is expected that they can improve myocardial function^14,15^.

#### Werner Otto Theodor Forssmann, André Cournard and Dickinson Richards – 1956: cardiac catheterization

The history of cardiac catheterization started in 1711, based on the works of Stephen Hales, who inserted tubes in both ventricles of a horse^1,16-19^. In the 19^th^ century, cardiac catheterization continued with the work of Claude Bernard, the father of modern physiology; it became more sophisticated with the skills of Chauveau and Marey; and started to be applied in the human heart thanks to the self‑confidence of Forssmann in 1929, becoming therapeutic in 1966^20^. According to Cournnand^17^, cardiac catheterization can be considered the "key that opened the lock to reveal the secrets of the heart." Due to the advancement of the technique proposed by the laureates, currently the hemodynamic study has applicability in the diagnosis, treatment and monitoring of cardiovascular disease - including Coronary Artery Disease (CAD) and HF.

The Nobel Prize in Physiology or Medicine in 1956 was awarded to three researchers, due to their researches on catheterization that revolutionized the studies on heart disease^1^. They would not accept with the scarcity of semiotic methods, of which provided diagnoses were rebuffed by autopsies, which served as motivation to initiate their studies^1^.

Werner Forssmann was a German physician, born in Berlin, who developed the hypothesis that a catheter could be inserted through blood vessels to the heart, aiming to injecting medications, perform contrast studies and measure chamber pressures. In order to test his hypothesis, he performed the first human catheterization in himself, guiding a catheter into his left atrium with the aid of a fluoroscopy device^1,16-19^.

Andre Cournard, born in Paris, France and Dickinson Richards, from Orange, United States, were physicians who worked in the development of the cardiac catheterization technique, with emphasis on pulmonary diseases and patterns of circulatory shock. They described the shock patterns, particularly cardiogenic and hemorrhagic (trauma) and analyzed the hemodynamic changes with treatment, either by fluid replacement or drug infusion^1,16-19^.

#### Frank Macfarlane Burnet and Peter Brian Medawar – 1960: immunological tolerance

Sir Frank Macfarlane Burnet was born in Australia and was the son of Scottish parents. He obtained a degree in Medicine in his native country and post-graduated in England. He specialized in the virology area, with important research related to influenza virus and herpes simplex. He played a key role in virus isolation from human tissue, and the first attempts to develop a vaccine for influenza. After World War II, he also developed researches related to the immune system, especially autoimmune mechanisms and immunological tolerance^1,2,21-23^.

**Figure 2:**
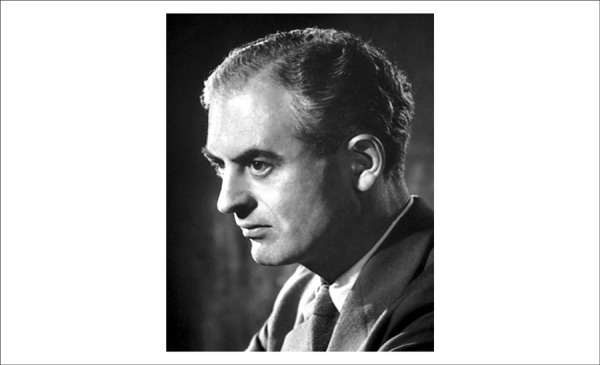
Peter Medawar’s picture.

Source: http://www.nobelprize.org/nobel_prizes/medicine/laureates/1960/medawar-facts.html

Peter Brian Medawar (Figure 2) was born in Petropolis (RJ), and had an English mother and Lebanese father. The family moved to England when he was only 14, where he developed his studies and career. Differences with the Brazilian government, which required his mandatory military service, made him give up his Brazilian nationality. He majored in zoology at Oxford and began transplantation research during World War II. The main objectives were skin grafts in burned skin areas. His studies led to the theory of acquired immunological tolerance, the basis for the development of solid organ transplants in the future^1,2,4,5^. The connection made between his research and Cardiology was the applicability of his results on immunological tolerance for the future development of heart transplantation.

#### Konrad Bloch and Feodor Lynen – 1964: cholesterol metabolism

The Nobel Prize in Physiology or Medicine of 1964 was awarded jointly to two German chemists, Konrad Bloch and Feodor Lynen. Bloch was born on January 21, 1912, in Neisse (now Nysa), formerly part of Germany and currently in Poland. He graduated in chemical engineering in 1934 in Munich. In 1936, due to the persecution of Jews by the Nazis, Bloch immigrated to the United States and joined the Department of Biochemistry at Columbia University, where he developed the research that led him to be awarded the Nobel Prize.

Feodor Lynen was born on April 6, 1911, in Munich, Germany, where he graduated in chemistry. He developed his entire career in Germany, living there even during the world wars^1,24^.

Even without performing a real joint work, both researchers carried out important discoveries in their universities on the cholesterol regulation mechanism and the fatty acid metabolism^1,24^. Starting from the idea that the acetic acid, with slow reaction in the chemical essays, had to show a more rapid and spontaneous reaction in the body, the concept of activated acetic acid was formulated, in which, in addition to adenyl-phosphoric acid as an energy source, also included coenzyme A. They were able to determine not only the structure of cholesterol, as well as the participation of coenzyme A in the oxidation of the fatty acids^1,24^. Years later, these discoveries were crucial to demonstrate the importance of cholesterol in atherosclerosis and, more importantly, helped the development of statins, the major class of drugs for treatment of hypercholesterolemia and atherosclerotic disease.

#### Allan M. Cormack and Godfrey Hounsfield - 1979: computed tomography

Allan Cormack was a South African biochemist and nuclear physicist born in Johannesburg (South Africa), who became a naturalized American in 1966. He is considered one of the inventors of computed tomography and shared the Nobel Prize in Physiology or Medicine with British professor Godfrey Hounsfield. Cormack performed his research in radiology initially at the University of Cape Town until he immigrated to the United States, where he worked at Harvard and Tufts University. There, he tested a mathematical model based on the X radiation, essential to the development of computed axial tomography^1,25^.

Sir Godfrey Hounsfield was an electrical engineer who had the position of "inventor" at the Central Research Laboratories in London. He started his career working on a radar project as a weapon of war and designed the first British transistorized computer in 1958, "EMIDEC 1100". Based on the mathematical calculations of radiation developed by Cormack, he developed Computed Tomography (CT) – so that the first machine to "scan" the brain was marketed by EMI. Three years later, he developed the first CT for the entire body. He continued to make improvements in CT and received numerous awards and honors, in addition to the Nobel prize, with his latest award being the title of Knight of the Queen of England - Sir, in 1981^1,25^. CT developed further in the following years and now has several applications in cardiology, for instance, determination of the coronary anatomy and the calcium score, and in the assessment of pulmonary thromboembolism.

#### Bengt Samuelsso, Sune Bergström and John Vane – 1982: prostaglandin function and development of angiotensinconverting enzyme inhibitors

Bengt Ingemar Samuelsso was a Swedish biochemist born in 1934 in Halmstad, a researcher at the Karolinska Institute in Sweden, and Nobel Prize laureate in Physiology or Medicine with his institute colleague and also Swedish biochemist Sune K. Bergström, and with the English chemist and pharmacologist John Robert Vane ^1^. His research involved prostaglandin function, purification, determination of their chemical structure and identification of their mechanism of formation from unsaturated fatty acids^1,26,27^. This information allowed the proposition of acetylsalicylic acid mechanism of action, practically indispensable treatment in coronary heart disease.

In addition to the research on prostaglandins, John Vane is also considered one of the "discoverers" of Angiotensin-Converting Enzyme inhibitors (ACE inhibitors)^1,26,27^. During the 1960s and 1970s, and with the participation of Brazilian Sergio Ferreira, Vane and his colleagues demonstrated key steps in the synthesis of angiotensin and bradykinin, which, in 1982, culminated in the launching of the first ACEI, captopril^1,26,27^. This class of drugs has a vital role in the treatment of hypertension, heart failure and coronary artery disease.

#### Michael Brown e Joseph Goldstein – 1985: LDL-cholesterol receptors

Joseph L. Goldstein was born on April 18, 1940 in Sumter, South Carolina, in the United States. He initially graduated in chemistry from Washington and Lee University and subsequently, obtained his medical degree at UT Southwestern Medical Center Dallas. He was a resident at Massachusetts General Hospital, where he met Michael S. Brown, who later would become his collaborator and together, they won the Nobel Prize^3^. During the two following years, he worked at the National Heart, Lung, and Blood Institute of the United States, which contributed to increase his skills and taste for scientific experimentation from the perspective of molecular biology in human disease^1,28-30^.

Michael Stuart Brown was born on April 13, 1941, in Brooklyn, New York. Similarly to Goldstein, he first obtained a degree in chemistry and only then in Medicine. He also worked at the National Heart, Lung, and Blood Institute of the US, but in the area of gastroenterology and hereditary diseases. In biochemistry laboratory, he learned enzymatic manipulation techniques, among which, an enzyme that could be related to familial hypercholesterolemia^1,28-30^.

Goldstein and Brown were awarded the Nobel Prize for scientific research in which they identified receptors on the surface of cells that mediate the uptake of Low-Density Lipoprotein (LDL) circulating in the bloodstream. Furthermore, they found that severe familial hypercholesterolemia is closely related to these receptors, as with the decrease in the number of membrane receptors, there is a lower uptake of circulating cholesterol in the form of LDL, thus increasing levels of the substance in the bloodstream from^1,28-30^. In this example, once again, one can observe the close association between high cholesterol levels, atherosclerosis, and ischemic heart disease.

#### James Black, Gertrude Elion and George Hitchings – 1988: beta-blockers

James W. Black, from Uddington, Scotland, studied medicine at the University of St. Andrews. In 1948, he started an investigation on cardiac adrenergic alpha and beta receptors, resulting in the synthesis of propranolol, the prototype of beta-blockers, essential medications for the treatment of heart failure and coronary artery disease. Later, in 1976, he concluded the synthesis of cimetidine, histamine H2-receptor antagonist, used in peptic disease treatments. Together with James W. Black, researchers Gertrude B. Elion (New York, United States) and George H. Hitchings (Hoquiam, United States) were also awarded the Nobel Prize of Medicine, for the development of drugs used in chemotherapy, antibiotics and antivirals^1,31^.

#### Joseph Edward Murray and Edward Thomas – 1990: organ transplantation

Joseph Murray was born on April 1^st^, 1919 in the city of Milford, State of Massachusetts (United States) and died in 2012 due to a stroke. He graduated from Harvard medical school and specialized in plastic surgery. He served in the US military and had a very important role in the care of wounded soldiers in World War II. When caring for burned patients, he observed that many patients responded well to donor skin grafts and decided to develop a research related to organ transplantation. On December 23, 1954, he was part of the team that made the first renal transplantation and, some years later, the first transplantation using a cadaveric source. Over the years, he participated in studies on immunosuppressive drugs such as azathioprine, aimed at reducing graft rejection^1,32^.

Edward Donall Thomas was born on March 20, 1920, in the city of Mart, State of Texas (United States), and also died in 2012. He graduated from medical school at Harvard and, early in his career, he devoted himself to laboratory studies related to bone marrow transplantation. Together with Joseph Edward Murray, he received the Nobel Prize in Physiology or Medicine as his studies helped to develop the transplantation of organs and tissues. In the field of HF, heart transplantation was indicated for patients that remained very symptomatic despite optimal medical treatment, which was performed for the first time in history in South Africa by Dr. Christiaan Barnard^1,32^.

#### Robert F. Furchgott, Ferid Murad and Louis J. Ignarro – 1998: nitric oxide

The Nobel Prize in Physiology or Medicine in 1998 was awarded jointly to Robert F. Furchgott (Charleston, United States), Louis J. Ignarro (New York, United States) and Ferid Murad (Whiting, United States) due to their findings on Nitric Oxide (NO) as a signaling molecule in the cardiovascular system^1^. NO is a soluble gas naturally found in the human body, which acts on the signaling of several biological processes.

The synthesis of NO occurs through the action of an enzyme called NO synthase (NOS) from L-arginine and L-citrulline amino acids, requiring for this enzymatic reaction, the presence of two cofactors, oxygen and Nicotinamide Adenine Dinucleotide Phosphate (NADPH). There are three types of NOS, two of them called constitutive and calcium-dependent NOS (cNOS), which are the endothelial and neuronal forms that synthesize NO in normal conditions and the calcium-independent form (iNOS), which is not expressed or is in a much lesser amount under physiological conditions. NO plays an important role in endothelial homeostasis, contributing with its vasodilating and anticoagulant properties. There is evidence that a decreased NO production is an important factor in ischemic events in patients with coronary artery disease and other suggesting that NO can exert antiatherosclerotic actions. Furthermore, the nitrates, the most widely used drugs in coronary artery disease and heart failure, act by indirectly increasing NO bioavailability^33-36^.

#### Paul C. Lauterbur and Peter Mansfield – 2003: magnetic resonance

Paul Lauterbur was born on May 6, 1929 in the United States, having obtained his PhD in Chemistry at the University of Pittsburgh in Pennsylvania (United States). Throughout his career has received numerous awards for his work related to magnetic resonance, including the gold medal of the Society of Magnetic Resonance in Medicine in 1982, the European Prize of Magnetic Resonance in 1986, the International Society for Magnetic Resonance in Medicine Award in 1992, the gold medal of the European Congress of Radiology in 1999, the NAS Award for Chemistry in Service to Society in 2001 and the Nobel Prize in Physiology or Medicine in 2003 together with Englishman Sir Peter Mansfield^1,37-39^.

Peter Mansfield was born on October 9, 1933 in London and a received his Ph.D. in physics in his hometown in 1962. In the course of his career as an investigator he received several awards, including the Gold Medal of the Society for Magnetic Resonance in Medicine in 1983, the European Prize of Magnetic Resonance in 1988, the title of Sir of the British Crown in 1993, the Gold Medal of the European Congress of Radiology and the European Association of Radiology in 1995 and the Nobel Prize for Physiology and Medicine, for his discoveries related to nuclear magnetic resonance imaging in 2003^1,37-39^.

Based on the initial findings of Lauterbur and Mansfield, MRI has developed and now has wide application in cardiology. It is considered, for instance, the gold standard in non-invasive myocardial and heart function assessment.

### Special Considerations

#### Carlos Chagas – 1911: an unjustly overlooked scientist in relation to the Nobel Prize

**Figure 3:**
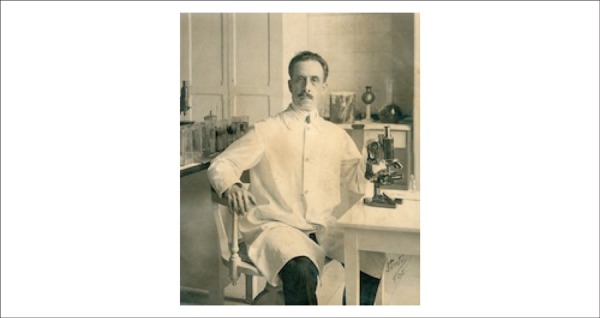
Carlos Chagas picture. Source: Lagoeiro B, Gemal P. Carlos Chagas. Um homem, uma doença, uma história. Niterói: Ed. UFF; 2012.

Carlos Chagas (Figure 3) was the first researcher in the world's scientific history to describe the complete cycle of a disease, currently known as Chagas disease^40^. His research with *Trypanosoma cruzi* started between the years 1907 and 1909, when he was sent to the countryside of the state of Minas Gerais to help fight malaria among workers building the Brazilian Central Railway^7^. In 1909, he identified the parasite in the blood of a child with "fever, anemia, edema and generalized lymphadenopathy" and later described the life cycle of* T. cruzi* ^7^. Aided by a small team, he changed history as a scientist that inspired a new era of knowledge, as he was able to draw a clinical profile from his own observations.

Throughout his career, he received honors of national importance, such as public health management positions, as well as international prizes, such as the Schauddinn award in Germany, for the most important discovery of protozoology. For these reasons, he was nominated four times for the Nobel Prize, but none fructified. Researchers and historians have assessed, among other sources, files from Oswaldo Cruz Foundation and the Karolinska Institute to identify the reasons why Carlos Chagas was not awarded the Nobel prize^6,7,41-43^.

Bestetti et al.^6,7,41,42^, in their set of historical publications, solidly based the reasons why Chagas was not a Nobel Prize laureate. These researchers even went to the Swedish institute in person to review the original documents of the time; their research, published in prestigious international journals, are less disseminated than they should be in Brazilian cardiology.

Gunnar Hedrèn was the board member of the Karolinska Institute who analyzed and issued an opinion on Chagas’ first nomination for the Nobel Prize in 1921^6,7^. Although there is no formal written opinion, the analysis of documents from that time suggests that the counselor did not value Chagas’ discovery^6,7^. Although now recognized as renowned researcher, Chagas had opponents in South America and Brazil. A group linked to the Bacteriology Institute in Buenos Aires, including a Brazilian member, insisted in the early years of Chagas' discovery, that there was no association between the symptoms reported by Chagas and the presence of* T. cruzi*^6,44^. Another group, from the very Oswaldo Cruz Foundation and the School of Medicine of Rio de Janeiro, disagreed with Chagas for political reasons and, on several occasions, questioned the importance of trypanosomiasis^6^. Among his opponents were Figueiredo de Vasconcellos, Cardoso Fontes and Plinio Marques^6^.

Chagas also lost prestige among the local population for unpopular measures at the time when he was appointed Director of Public Health, an equivalent post at the time of the "Minister of Health". The mandatory vaccine for smallpox was one of those most criticized measures, being even the reason of a popular revolt^6,42^. Finally, the methods used by the then President of the Karolinska Institute, JE Johansson, are criticized. It is suggested that he excessively valued researches related to physiology, rather than those related to clinical medicine^42^.

A new indication and a Nobel award after Chagas’ death are no longer expected. It persists, though, in the light of the centenary of the discovery by Chagas, our pride in a great Brazilian who, with all the honors and merits, made such a contribution to humankind.

#### Bernard Lown – a brilliant clinical cardiologist that received the Peace Nobel Prize of 1985

Bernard Lown was born on June 7, 1921, in the city of Utena, Lithuania, and moved at age 13 with his family to the USA and settled in the state of Maine, where he became a medical doctor and completed his specialization in cardiology at the current Brigham and Women's hospital in Boston^1,45^. Together with engineer Baruch Berkowitz, in 1961, he created the direct current used in the defibrillator, allowing greater safety and efficacy in relation to the then AC defibrillator created by Paul Zoll. Lown also discovered the correct moment of the cardiac cycle in ECG for the electrical discharge in ventricular tachyarrhythmias. This therapy received the name of "cardioversion". The defibrillator designed by Lown and Berkowitz was used as standard therapy in cardiac arrhythmias until the 1980s, when the models with biphasic current were created. He also has researched the use of lidocaine as an antiarrhythmic drug and the importance of serum potassium in digitalis intoxication. Lidocaine, until then, was basically used as a local anesthetic by dentists. In the presence of HF, electrical therapies are essential in preventing sudden death (of which ventricular dysfunction is one of the most important risk factors) and in the treatment of symptomatic arrhythmias, highly prevalent in this group; digoxin is one of the drugs indicated for patients with reduced ejection fraction and symptomatic ones with functional class III or IV^1,45^.

Despite all these contributions to Medicine, his Nobel Prize was won by other merits: a peacekeeper, he created the International Physicians for the Prevention of Nuclear War, in association with the then Soviet citizen Yevgeniy Chazov. His association has also had the participation of Brazilian physicians. He also published two famous books: “The lost art of healing” and “Prescription for survival: a doctor's journey to end nuclear madness”. He is currently a Professor Emeritus at Harvard University and founder of the Lown Institute, of which mission is described on their website as: “to help set up a sustainable and compassionate health system, where doctors can serve as healers and lawyers, where patients receive the services they need and are protected from unnecessary treatment and damage, and where financial incentives are removed from clinical decision-making”^1,45^.

## Conclusion

The Nobel Prize aims to reward researchers whose actions and discoveries have contributed exceptionally to the progress and the good of society. Regarding heart failure, the final pathway of several forms of heart disease, 33 researchers in 16 awards performed studies that yielded great contributions to its diagnosis and treatment. Brazil, despite its growing scientific contributions in recent decades, in the fields of Physiology and Medicine, has no "genuinely" Brazilian laureates, despite the contributions of Dr. Peter Medawar and Dr. Carlos Chagas - the latter unjustly not a recipient of this honor.
